# Towards a minimal EEG channel array for a biometric system using resting-state and a genetic algorithm for channel selection

**DOI:** 10.1038/s41598-020-72051-1

**Published:** 2020-09-10

**Authors:** Luis Alfredo Moctezuma, Marta Molinas

**Affiliations:** Department of Engineering Cybernetics, Norwegian University of Science and Technology, 7491 Trondheim, Norway

**Keywords:** Applied mathematics, Computational neuroscience, Computational models, Data processing, Machine learning, Biomarkers

## Abstract

We present a new approach for a biometric system based on electroencephalographic (EEG) signals of resting-state, that can identify a subject and reject intruders with a minimal subset of EEG channels. To select features, we first use the discrete wavelet transform (DWT) or empirical mode decomposition (EMD) to decompose the EEG signals into a set of sub-bands, for which we compute the instantaneous and Teager energy and the Higuchi and Petrosian fractal dimensions for each sub-band. The obtained features are used as input for the local outlier factor (LOF) algorithm to create a model for each subject, with the aim of learning from it and rejecting instances not related to the subject in the model. In search of a minimal subset of EEG channels, we used a channel-selection method based on the non-dominated sorting genetic algorithm (NSGA)-III, designed with the objectives of minimizing the required number EEG channels and increasing the true acceptance rate (TAR) and true rejection rate (TRR). This method was tested on EEG signals from 109 subjects of the public motor movement/imagery dataset (EEGMMIDB) using the resting-state with the eyes-open and the resting-state with the eyes-closed. We were able to obtain a TAR of $1.000 \pm 0.000$ and TRR of $0.998 \pm 0.001$ using 64 EEG channels. More importantly, with only three channels, we were able to obtain a TAR of up to $0.993 \pm 0.01$ and a TRR of up to $0.941 \pm 0.002$ for the Pareto-front, using NSGA-III and DWT-based features in the resting-state with the eyes-open. In the resting-state with the eyes-closed, the TAR was $0.997 \pm 0.02$ and the TRR $0.950 \pm 0.05,$ also using DWT-based features from three channels. These results show that our approach makes it possible to create a model for each subject using EEG signals from a reduced number of channels and reject most instances of the other 108 subjects, who are intruders in the model of the subject under evaluation. Furthermore, the candidates obtained throughout the optimization process of NSGA-III showed that it is possible to obtain TARs and TRRs above 0.900 using LOF and DWT- or EMD-based features with only one to three EEG channels, opening the way to testing this approach on bigger datasets to develop a more realistic and usable EEG-based biometric system.

## Introduction

The number of biometric systems based on electroencephalography (EEG) has been steadily growing using various approaches to solve problems related to the authentication and verification stages. A research-grade EEG device guarantees a controlled environment and high-quality multi-channel EEG recording, which is offset by the high computational cost, non-portability of the equipment, and use of inconvenient conductive gels. The development of dry EEG sensors has created new possibilities for the development of new types of portable EEG systems.

An important step towards this goal is a reduction in the number of required EEG channels while increasing, or at least maintaining, the same performance high-density EEGs. Depending on whether the paradigm is task-related or task-free, certain EEG channels provide only redundant or sub-optimal information. Several techniques have been studied with the aim of developing low-density EEG-based systems with high performance, i.e, pre-processing and feature extraction, channel selection, and paradigms to stimulate brain signals. For EEG-based biometric systems, several approaches have been presented using various paradigms to stimulate and record the EEG signals, i.e. imagined speech^1–3^, resting-state^4–10^, and event-related potentials (ERPs)^11,12^.

The use of the resting-state does not require any training before using the system. We present an approach using the resting-state during which the eyes of the subjects were open and repeated the experiments using the resting-state during which the eyes of the subjects were closed. We organized our approach as a multi-objective optimization problem with three unconstrained objectives: (1) reduce the necessary number of EEG channels to perform identification, (2) obtain a true acceptance rate (TAR) as high as possible, and (3) obtain a true rejection rate (TRR) as high as possible. The TAR is the percentage of times the system correctly verifies a true claim of identity, and the TRR the percentage of times it correctly rejects the subjects that are not in the system.

In general, there are a number of approaches that use raw data as input for various configurations of neural networks (NN)^13–16^ and several have been proposed to tackle the high dimensionality of the data using methods for feature extraction, such as principal component analysis (PCA)^17^, the discrete wavelet transform (DWT)^3^, or empirical mode decomposition (EMD)^2,3,11,12^.

Several approaches have been proposed for the creation of a biometric system following various experiment configurations, with various paradigms and methods for feature extraction and classification using the public EEG Motor Movement/Imagery Dataset (EEGMMIDB), using various configurations of neural networks^14,18–20^, other supervised and unsupervised techniques^5,21–31^, and methods for EEG channel selection^6,32,33^.

One approach used a subset of eight pre-selected channels^32^ and EEG data from a task for training and then that from another task for testing. The selection of the channels was justified based on their stability across various mental tasks, and the results presented were evaluated using the half total error rate (HTER), which was 14.69%. Another approach used various tasks from the EEGMMIDB and channel selection, using the binary flower pollination algorithm (BFPA),and reported accuracies of up to 0.87 using supervised learning and approximately 32 EEG channels^33^. However, the analysis considered only non-intruders when using multi-class classification, and therefore the addition of more stages for detecting the intruders is necessary.

Other approaches use instances of different length with the same dataset, such as instances of 10 or 12 s^5,24^. Resting-state instances of 10 s have been validated with the leave one-out framework, consisting of five instances of 10 s for training and one instance for validating the model^24^, resulting in a correct recognition rate (CRR) of 0.997 for the resting-state with the eyes-open, and 0.986 with the eyes-closed, all using 64 EEG channels. Biometric systems based on resting-state and one-second EEG signals from only two channels (FP1 and FP2) and a 256-Hz sample rate has been proposed by extracting features directly from the raw data and using Fisher’s discriminant analysis, obtaining an up to 0.966 TAR and a 0.034 FAR^9^.

Deep learning algorithms have shown to be a success in image processing and other fields, but when using EEG data they have not shown convincing and consistent improvements over the most advanced methods to date^16,34^. However, recently some new approaches have been presented with high accuracies, for instance, an approach using Convolutional Neural Networks (CNN) Gated Recurrent Unit (CNN-GRU) was presented^15^, and the authors evaluated the proposed method in a public dataset called DEAP, which consists of EEG signals from 32 subjects using 32 channels using different emotions as a paradigm^35^. Their experiments were performed using 10-s segments of EEG signals and reported that CNN-GRU reached up to 0.999 mean CRR with 32 channels, and 0.991 with 5 channels that were selected using one-way repeated measures ANOVA with Bonferroni pairwise comparison (post-hoc). The findings of this work are interesting and the accuracies obtained are high. However, deep learning approaches need to use a large amount of data, as well as the length of the signal segments and the paradigm followed are not the standard, and for a real-time application, the collection of a large number of instances, and instances during long periods can be exhausting and not competitive with current biometric systems in the industry (i.e fingerprint, face recognition, etc.).

The amount of data and time required for training NN are the main concerns for effective deployment and adoption in real-life scenarios of EEG-based biometric systems. In the literature, researchers report from simple NN structures (i.e., a single hidden layer) to more complex networks (Recurrent and CNN), but it requires the improvement of computational power, with faster CPUs and the use of GPUs^15,28–31,34^. To overcome the need for a large amount of data of deep learning approaches, there is an approach that uses simple data augmentation techniques by creating overlapped time windows^18^. Other related proposals using neural networks have been presented and compared in the state of the art^28–31^, where some of the most relevant works used around 100 subjects and in most of the cases 64 channels for testing their approaches^13,14,18,36^. However, there is no defined method for channel selection, since the process for selecting the most relevant channels requires the repetition of the classification process several times, and it is well known that deep learning approaches are computationally costly^30,34^.

Based on the results of our previous studies^2,7,12^, we compared EMD and DWT for decomposing the EEG signals into different frequency bands. When using EMD, we selected the two sub-bands closest to the original EEG signal for each channel, and for DWT we decomposed the EEG signal into four different sub-bands (Three levels of decomposition and the residual), as explained in the “Methods” section. For each sub-band, we then extracted the instantaneous and Teager energy and the Higuchi and Petrosian fractal dimensions, thus obtaining a set of eight features per channel when using EMD and 16 features for DWT.

We present the results from a set of experiments using the EEGMMIDB dataset, with 109 subjects, for creating an EEG-based biometric system that creates a model for each subject and can reject the other 108. We also present results from experiments using one-class support vector machine (OC-SVM) with the *radial basis function (RBF)* to compare the behavior with the second approach, which uses the local outlier factor (LOF). All the experiments were validated using 10 runs with 80% of the instances for training and 20% for testing. We solved the optimization problem using the non-dominated sorting genetic algorithm (NSGA)-III, which uses the Pareto-optimal solutions and a niche method to maintain stable sub-populations of good points^37^. NSGA-III has proven to be a robust method for channel selection, especially when using more than two objectives^12,38^. First, we report the results for a set of experiments using all the available channels to show the TAR and TRR using different parameters. We then present the results for experiments using OC-SVM and the optimization process with EEG signals from the resting-state in which the eyes of the subjects were open. Finally, we repeated the experiments, solving the optimization problem for channel selection using LOF with different parameters and the EEG signals from the resting-state in which the eyes of the subjects were open and then closed.

## Results

### Subject identification using 64 EEG channels, comparing OC-SVM and LOF using different parameters

We used EEG signals from 64 channels of 109 subjects and 60 instances of one second with a sample rate of 160 Hz that were recorded during the resting-state in which the eyes of the subject were open. We used EMD- or DWT-based features, and evaluated the results using the TAR and TRR.

For ensuring tenfold cross-validation, we performed the experiments 10 times, selecting randomly 80% of the instances for training and 20% for testing, in this way we are ensuring that the method can be generalized and that the results can be obtained even using another subset of instances for training and testing. The models were created using OC-SVM or LOF models. It should be noted that the channels and parameters were optimized for all the subjects at the same time but creating a single machine-learning model for each subject. In general, the results presented in Table 1 were obtained by creating a model for each of the 109 subjects in which the model of the subject was used to recognize the subject and reject the rest of the 108 that were not part of the model.

**Table 1:** Average TARs and TRRs for subject detection with EEG data from 64 channels using different parameters for OC-SVM and LOF and DWT-based features with 109 subjects.

Method	Algorithm	No. neighbors	EMD		DWT	
			TAR	TRR	TAR	TRR
OC-SVM			$0.502 \pm 0.004$	$0.993 \pm 0.001$	$0.499 \pm 0.002$	$0.998 \pm 0.000$
LOF	Ball tree	1	$1.000 \pm 0.000$	$0.923 \pm 0.005$	$1.000 \pm 0.000$	$0.979 \pm 0.002$
LOF	Ball tree	10	$0.926 \pm 0.002$	$0.963 \pm 0.007$	$0.968 \pm 0.0038$	$0.989 \pm 0.012$
LOF	kd tree	1	**1**.**000** ± **0**.**000**	**0**.**989** ± **0**.**005**	**1**.**000** ± **0**.**000**	**0**.**998** ± **0**.**001**
LOF	kd tree	10	$0.926 \pm 0.001$	$0.955 \pm 0.006$	$0.923 \pm 0.001$	$0.988 \pm 0.002$
LOF	Brute	1	$1.000 \pm 0.000$	$0.926 \pm 0.004$	$1.000 \pm 0.000$	$0.979 \pm 0.004$
LOF	Brute	10	$0.927 \pm 0.001$	$0.939 \pm 0.007$	$0.924 \pm 0.003$	$0.989 \pm 0.002$

Bold values indicates the best relationship between TAR and TRR.

The results obtained with OC-SVM showed the lowest TAR (Table 1), meaning that the models created with OC-SVM did not learn from the training set and thus rejected approximately 50% of the instances, on average, explaining why the TRR was high when using OC-SVM. The results obtained with LOF, using three different algorithms and one or ten neighbors, are also shown in Table 1 for illustrative purposes. LOF using the *k-d tree* algorithm and one neighbor resulted in the highest TAR and TRR, meaning that it was possible to identify each subject and reject almost all the rest that did not correspond to the models.

**Figure 1:**
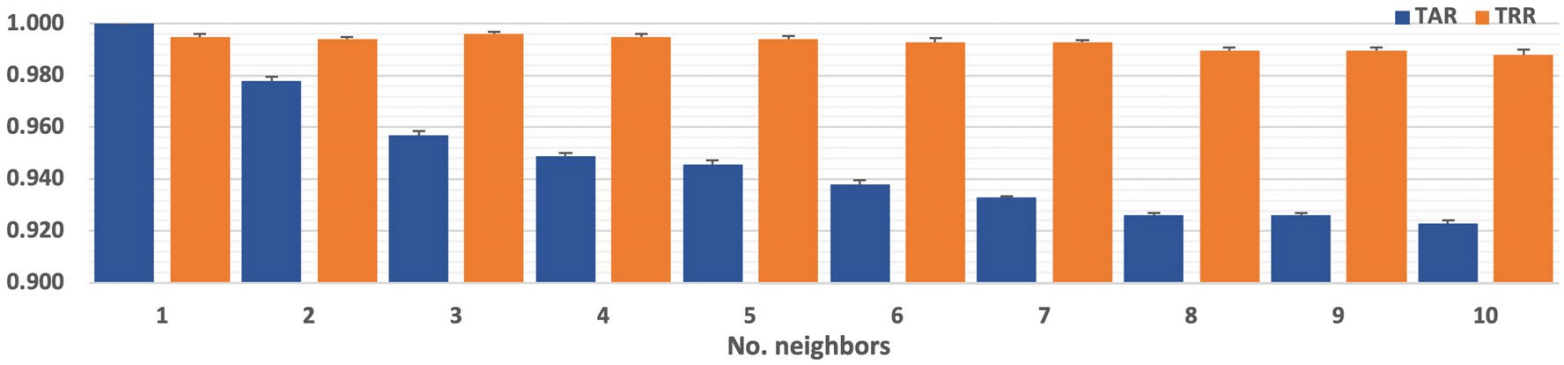
TARs and TRRs obtained using various numbers of neighbors with the LOF *k-d tree* algorithm and DWT-based features.

Previous results have shown that the algorithm and number of neighbors used are important for increasing the TAR and TRR. We repeated the experiments using DWT-based features considering only LOF with the *k-d tree* and 1 to 10 neighbors to provide more information about this behavior The average results obtained using 10-fold cross-validation are presented in Fig. 1.

The use of a higher number of neighbors resulted in a decrease in the TAR from 1.000 to 0.923 and an increase in the TRR or its remaining higher than 0.988 (Fig. 1), meaning that the models were unable to learn about the features of each subject using that number of neighbors. This is relevant, as it shows the importance of selecting not only the best feature extraction method but also the LOF algorithm and the best number of neighbors.

### Channel selection using NSGA-III and OC-SVM for EEG signals from the resting-state with the eyes open

We previously showed that the TAR and TRR of the models created using OC-SVM can be improved by finding the best *nu* and *gamma* parameters^12^. We performed the optimization process defined in the “Methods” section to provide more information about the behavior of OC-SVM models using a bigger dataset, trying to improve the TAR and TRR while reducing the necessary number of EEG channels for subject identification.

For this experiment, we used EEG signals of the 109 subjects during the resting-state with their eyes-open, using 80% of the instances for training and 20% for testing. We used NSGA-III for the channel selection method using 64 binary genes in a chromosome to represent the EEG channels (1 if the channel is used, 0 if not) and two genes with decimal values (both from 0 to 1) to select the best *nu* and *gamma* parameters, obtaining thus a chromosome of 66 genes.

**Figure 2:**
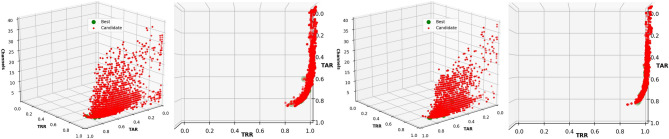
Frontal and aerial view of the TARs and TRRs obtained in the channel selection process using EMD-based features (left) and DWT-based features (right) with OC-SVM.

The distribution of the results of one run, as an example, obtained using EMD- and DWT-based features is shown in Fig. 2. The average and standard deviation of the results obtained using 10-fold cross-validation are presented in Table 2.

**Table 2:** TARs and TRRs obtained for the first five EEG channels in the Pareto-front for three objectives solved with NSGA-III using EMD- and DWT-based features with OC-SVM.

No. channels	EMD		DWT	
	TAR	TRR	TAR	TRR
1	$0.776\pm 0.138$	$0.851\pm 0.055$	$0.801\pm 0.063$	$0.905\pm 0.042$
2	$0.776\pm 0.092$	$0.911\pm 0.043$	$0.774\pm 0.066$	$0.958\pm 0.023$
3	$0.763\pm 0.150$	$0.969\pm 0.020$	$0.629\pm 0.180$	$0.959\pm 0.022$
4	$0.779\pm 0.144$	$0.966\pm 0.033$	$0.720\pm 0.069$	$0.980\pm 0.020$
5	**0**.**822 **± **0.028**	**0**.**969** ± **0**.**022**	**0**.**822** ± **0**.**028**	**0**.**981** ± **0**.**017**

Bold values indicates the best relationship between TAR and TRR.

As mentioned previously, we performed the optimization 10 times for cross-validation. For some runs, the Pareto-front contained only channel combinations with one to five channels and others with one to seven. Using these different subsets found, we can further analyze the channels in common, and the others. With this, it may be possible to recommend a set of channels for a new possible headset (Considering the best subset found and the most appropriate for a new design.), but first, it is necessary to perform the analysis to choose the best paradigm or sub-task (i.e. resting-state with the eyes open or closed) for EEG data collection. For comparative purposes, we present the average TAR and TRR obtained using channel combinations of one to five channels in the Pareto-front of the 10 runs.

We obtained a TAR of $0.822 \pm 0.028$ and a TRR of $0.969 \pm 0.022$ with only five channels when using EMD-based features (Table 2). The TAR and TRR were $0.822 \pm 0.028$ and $0.981 \pm 0.017,$ respectively, when using DWT-based features and five channels with the optimization process.

As it is presented in Fig. 2, the candidates generated using EMD- or DWT-based features and OC-SVM showed a clear tendency to reject all the subjects (Which increases the TRR, since the models are rejecting correctly the intruders), even the subject in each model (Which decreases the TAR), meaning that the models created for each subject did not learn from the provided features. TAR increases only if the right *nu* and *gamma* parameters and channels are selected, which also vary in each run and is reflected in the standard deviation.

**Figure 3:**
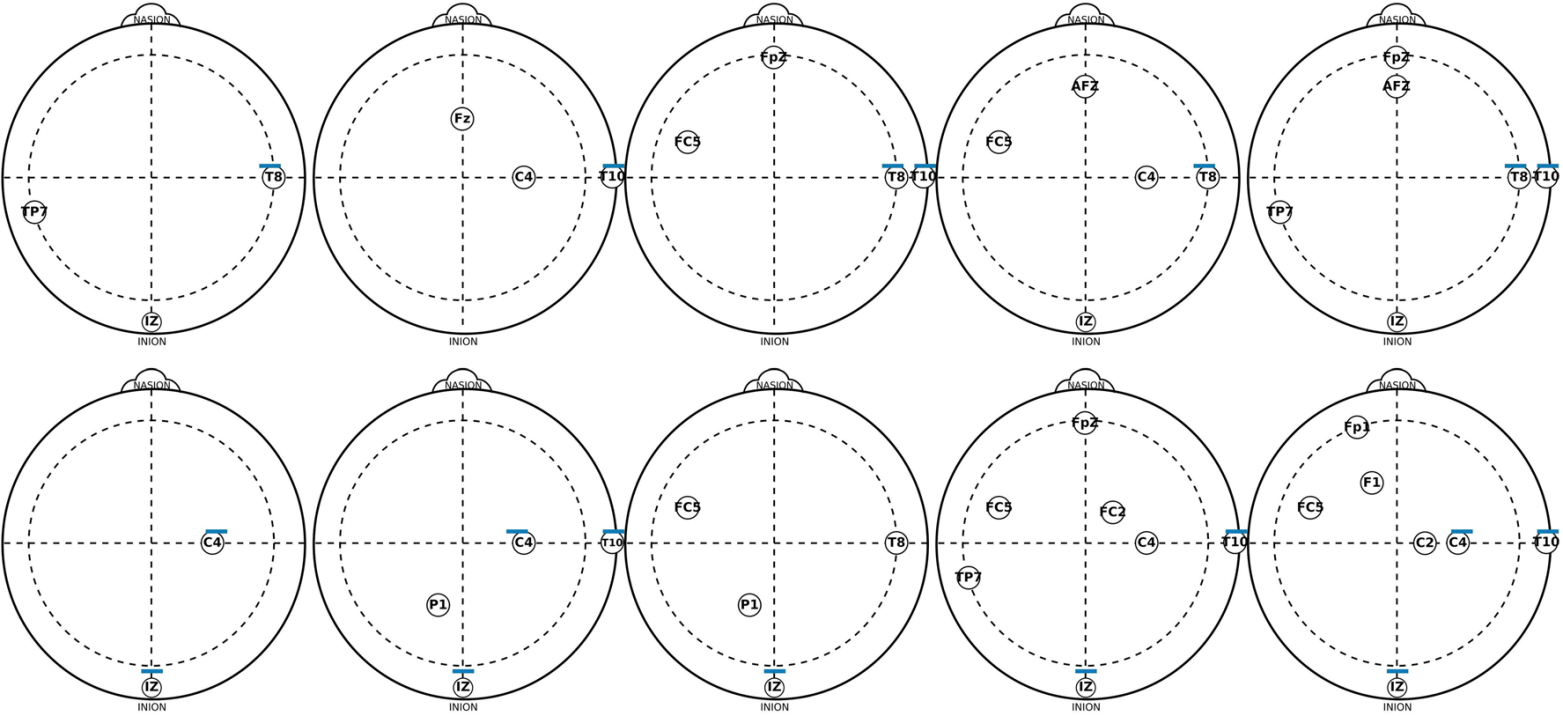
Set of one to five channels found during the optimization process for creating the biometric system with OC-SVM using EMD-based features (top) or DWT-based features (bottom), and resting-state with the eyes open.

Figure 3 presents the set of channels used during the optimization process in the 10 runs. The set of channels found when using EMD-based features are presented in the top and the bottom is for DWT-based features. Each set of channels from left to right correspond to using one to five channels, and as it was mentioned early, in some of the runs, the channels found by NSGA-III were different to other runs, but the figure presents one set of them. Using EMD-based features, the channels found when using one to five channels differ, but it is possible to observe that channels around *T10* and *T8* are consistent across most sets. When using DWT-based features it is more clear that channel *IZ* appears in all the sets, and channels *C4* and *T10* appear in most of the sets.

### Channel selection using NSGA-III and LOF for EEG signals from the resting-state with the eyes open

We performed the optimization process using the 109 subjects in the dataset, but now considering LOF for creating the models of each subject. We used NSGA-III for the channel selection method using 64 binary genes in a chromosome to represent the EEG channels and two genes with integer values to select the algorithm (1: Ball tree, 2: k-d tree, 3: Brute force) and the number of neighbors (From 1 to 10, which were proposed experimentally) to be used, obtaining thus a chromosome of 66 genes. The experiment was repeated 10 times for validation, each time using 80% of the instances of each subject for training and 20% for testing.

**Figure 4:**
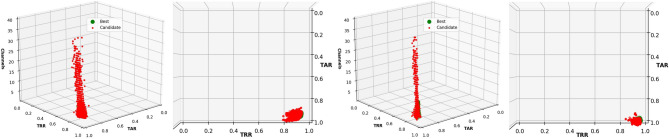
Frontal and aerial view of the TARs and TRRs obtained in the channel selection process using EMD-based features (left), and DWT-based features (right) with LOF.

The results of the first run are presented in Fig. 4 as an example of the distribution of the TARs and TRRs during the optimization process and Table 3 presents the average results for both methods of feature extraction, EMD or DWT.

**Table 3:** TARs and TRRs obtained for the first seven EEG channels in the Pareto-front for three objectives solved with NSGA-III using EMD-based features and LOF.

No. channels	EMD		DWT	
	TAR	TRR	TAR	TRR
1	$0.930\pm 0.005$	$0.904\pm 0.006$	$0.979\pm 0.001$	$0.888\pm 0.003$
2	$0.949\pm 0.002$	$0.909\pm 0.005$	$0.991\pm 0.001$	$0.922\pm 0.002$
3	$0.960\pm 0.003$	$0.909\pm 0.005$	**0**.**993 **± **0**.**001**	**0**.**941 **± **0**.**002**
4	$0.964\pm 0.005$	$0.918\pm 0.028$	$0.995\pm 0.011$	$0.949\pm 0.004$
5	$0.969\pm 0.008$	$0.926\pm 0.011$	$0.996\pm 0.006$	$0.952\pm 0.004$
**6**	$0.980\pm 0.003$	$0.938\pm 0.011$	$0.997\pm 0.006$	$0.957\pm 0.009$
**7**	$0.980\pm 0.004$	$0.940\pm 0.005$	$0.997\pm 0.001$	$0.957\pm 0.005$

Bold values indicates the best relationship between TAR and TRR with the lowest number of channels.

**Figure 5:**
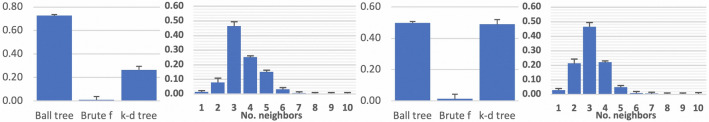
Average distribution of the algorithms and number of neighbors used in the optimization process with EMD-based features (left) and DWT-based features (right).

**Figure 6:**
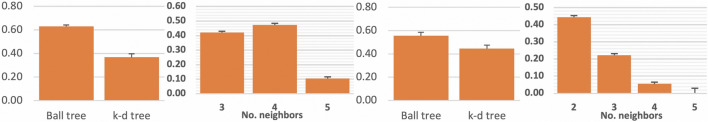
Average distribution of the algorithms and number of neighbors used for the results in the Pareto-front of the optimization process with EMD-based features (left) and DWT-based features (right).

The use of DWT-based features allowed us to obtain an average TAR of up to $0.993 \pm 0.001$ and a TRR of $0.941 \pm 0.002$ using only three EEG channels (Table 3). Interestingly, the distribution of the results was very distinct and clear (Fig. 4), indicating that we can obtain similar TARs and TRRs with different channel combinations using LOF and EMD- or DWT-based features.

The average distribution of the parameters used in the complete optimization process (In all generations and all chromosomes) is presented in Fig. 5, showing that the algorithm more often used by LOF was *ball tree* with three neighbors when using EMD-based features. The *ball tree* and *k-d tree* algorithms were used equally, with three neighbors, when DWT-based features were used. Analysis of only the parameters used for the results in the Pareto-front in the 10-fold cross-validation (For obtaining the results presented in Table 3) confirmed that the *ball tree* algorithm with 3 to 4 neighbors is the most often used for EMD-based features, and for DWT-based features, the *ball tree* and *k-d tree* are used with only two neighbors, as it is shown in Fig. 6.

**Figure 7:**
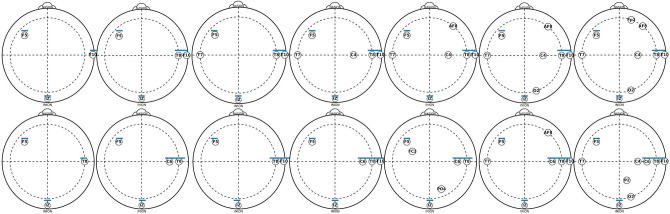
Set of one to seven channels found during the optimization process for creating the biometric system with LOF, and EMD-based features (top) or DWT-based features (bottom), and resting-state with the eyes open.

Figure 7 presents the set of channels of the 10 runs used for obtaining the results on Table 3, which correspond to the use of one to seven channels, using EMD-based features (Top of the figure) and DWT-based features (Bottom of the figure). Interestingly, in this case, the channels are almost the same using both methods, and they did not differ too much when using one or three channels. Another important point is that channels *IZ, T8*, and *T10*, appear in most of the cases, in both methods, using EMD- or DWT-based features. It is possible to observe that the most relevant area is around *C6, T8, T10* and *F5* channels.

### Channel selection using NSGA-III and LOF for EEG signals from the resting-state with the eyes closed

Previous experiments using LOF resulted in higher TARs and TRRs with a lower number of EEG channels than using OC-SVM. We repeated the optimization process with EEG data from 109 subjects but considering the resting-state with the eyes closed, as described in the “Methods” section, to provide additional information about the performance of LOF with EMD- or DWT-based features.

**Figure 8:**
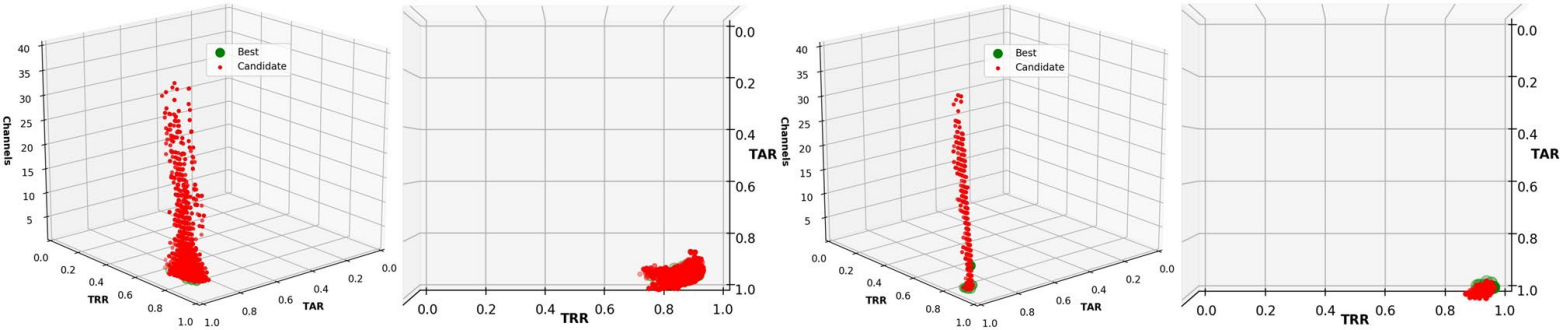
Frontal and aerial view of the TARs and TRRs obtained in the channel selection process using EMD- (Left) and DWT-based features (Right) from the resting-state with the eyes closed, using LOF.

The chromosome representation was as in the previous experiment: 64 genes for representing the EEG channels and two additional genes with integer values for the different algorithms and number of neighbors. Each experiment was performed 10 times, randomly selecting 80% of the instances for training and 20% for testing, ensuring thus the tenfold cross-validation. The results obtained from a run when using EMD- and DWT-based features are presented in Fig. 8 for visualization and understanding the behavior during the optimization process.

**Table 4:** TARs and TRRs obtained with LOF for the first seven EEG channels in the Pareto-front for three objectives solved with NSGA-III, using EMD- or DWT-based features and the resting-state with the eyes closed.

No. channels	EMD		DWT	
	TAR	TRR	TAR	TRR
1	$0.945\pm 0.005$	$0.888\pm 0.008$	$0.979\pm 0.001$	$0.881\pm 0.004$
2	$0.945\pm 0.005$	$0.918\pm 0.007$	$0.995\pm 0.001$	$0.935\pm 0.005$
3	$0.955\pm 0.005$	$0.918\pm 0.007$	**0**.**997**± **0**.**002**	**0**.**950**± **0**.**005**
4	$0.969\pm 0.003$	$0.926\pm 0.006$	$0.997\pm 0.002$	$0.950\pm 0.003$
5	$0.971\pm 0.002$	$0.933\pm 0.002$	$0.997\pm 0.002$	$0.951\pm 0.003$
6	$0.975\pm 0.001$	$0.945\pm 0.002$	$0.998\pm 0.000$	$0.953\pm 0.002$
7	$0.979\pm 0.002$	$0.955\pm 0.005$	$0.998\pm 0.000$	$0.955\pm 0.002$

Bold values indicates the best relationship between TAR and TRR with the lowest number of channels.

**Figure 9:**
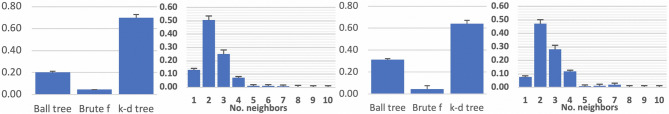
Average distribution of the algorithms and number of neighbors used in the optimization process with EMD-based features (left) and DWT-based features (right) using EEG signals from the resting-state with the eyes closed.

**Figure 10:**
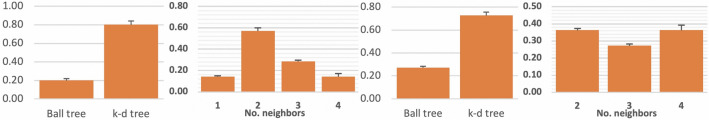
Average distribution of the algorithms and number of neighbors used for the results in the Pareto-front of the optimization process with EMD-based features (left) and DWT-based features (right) using EEG signals from the resting-state with the eyes closed.

The average TAR and TRR in the Pareto-front for the first seven channels, in both cases, using EMD and DWT for feature extraction, are presented in Table 4. The results show that subject identification is possible using the resting-state with the eyes closed. The TAR and TRR are similar to those presented in Table 3. Interestingly, the results were maintained in the 10 runs, especially when using DWT for feature extraction, as the standard deviation was 0.011 for the TAR and 0.009 for the TRR.

The average distribution of the parameters used during the entire optimization process is shown in Fig. 9. The *k-d tree* algorithm was the most used, in both cases, using EMD or DWT, and the number of neighbors ranged from 1 to 4, with a clear advantage of using two neighbors. The average parameters used for obtaining the results in the Pareto-front are presented in Fig. 10, confirming that the *k-d tree* algorithm is the most used and the number of neighbors still ranges from 1 to 4, with preferential use of only 2 neighbors.

**Figure 11:**
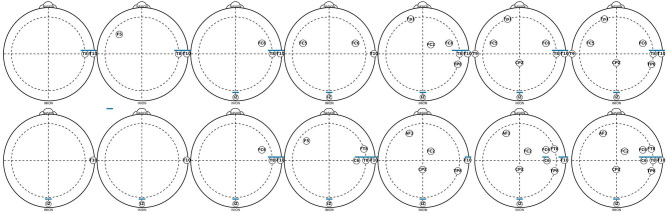
Set of one to seven channels found during the optimization process for creating the biometric system with LOF, using EMD-based features (top) or DWT-based features (bottom), and resting-state with the eyes closed.

As in the previous experiment using resting-state with eyes open, Fig. 11 presents the set of channels found in the optimization process of the 10 runs creating the models for the biometric system using resting-state with the eyes closed, using EMD-based features (Top of the figure) as well as DWT-based features (Bottom of the figure). Interestingly, the results presented in Figs. 7 and 11 do not differ too much, even across methods and in the sets of different number of channels (In the sets created in the 10 runs with 1 to 7 channels). The most relevant area is still around the channels *C6, T8, T10* and *IZ*.

## Discussion

Our research on biometric systems has focused on the study and comparison of various task-related and task-free paradigms, i.e. resting-state, ERPs, and imagined speech, using various types of electrodes, and various numbers of channels^2,3,7,11^. The resting-state has been used in the state-of-the-art for this purpose since it does not require any training process for the subject. There are approaches based on multi-class classification using machine-/deep-learning and one-class classification. Although most of the approaches can discriminate between the subjects in the database when using multi-class classification, they do not consider possible intruders. In the best case, a study presented a set of eight EEG channels selected beforehand^32^. Another study used deep learning with a set of five EEG channels also selected beforehand, but they did not use resting-state^15^. We previously presented a method for channel selection using a two-stage method tested on a dataset with 26 subjects for detecting intruders and then using multi-class classification to detect the name of the subject^12^. The stage for intruder detection was created using OC-SVM with *nu* and *gamma* parameters determine by a genetic algorithm that also selected the most relevant channels for the task. However, OC-SVM has been shown to be very sensitive to the *nu* and *gamma* parameters.

Here, we present a new approach for an EEG-based biometric system using brain signals recorded during the resting-state with the eyes open and the resting-state with the eyes closed, using LOF and channels selected by NSGA-III. In short, we created a model using LOF with EMD-/DWT-based features for each subject, and then were able to reject the other 108 subjects in the dataset, confirming that the features extracted from each subject can help to discriminate between the subject in the model and the rest of the subjects, with good results, even with a low number of EEG channels and using 108 subjects as intruders.

First, we conducted experiments using EEG signals from the resting-state with the eyes open and 64 EEG channels, with OC-SVM and LOF, using different parameters. We show that we can attain a TAR of up to $1.000 \pm 0.000$ and a TRR of $0.998 \pm 0.001$ using LOF and the *k-d tree* algorithm with only one neighbor, all using DWT-based features. Then, we repeated the experiment using 1 to 10 neighbors with DWT-based features, LOF, and the *k-d tree* algorithm, as they were the best parameters found in the previous experiment, and also to show that a different number of neighbors affects the TAR and TRR.

We also showed that OC-SVM resulted in a TAR of $0.502 \pm 0.004$ and a TRR of $0.993 \pm 0.001,$ meaning that the models are unable to learn from any of the features of the subject (EMD- or DWT-based). It was thus necessary to fit the best *nu* and *gamma* parameters by using the multi-objective optimization process^12^. This resulted in substantially higher TAR and TRR values (See Fig. 2). In the best case, we obtained a TAR of up to $0.822 \pm 0.028$ and a TRR of $0.969 \pm 0.22$ with EMD-based features, and a TAR of $0.822 \pm 0.28$ and a TRR of $0.981 \pm 0.017$ with DWT-based features. However, the standard deviation was high.

The results presented with LOF when using the resting-state with the eyes open show that we can obtain a TAR of up to $0.993 \pm 0.01$ and a TRR of $0.941 \pm 0.002,$ with only three EEG channels, and with only two EEG channels using DWT-based features. We obtained a TAR and TRR above 0.900, which are higher than the best results obtained in the Pareto-front using EMD-based features. As shown in Fig. 4, the distribution of the TAR and TRR values was consistent when reducing the number of EEG channels during the optimization process, showing that the models created with LOF learn well from the features provided and that different channel combinations are used to obtain the best results, as presented in Table 3. In this case, the most used algorithm for the complete optimization process was *ball tree*, with three neighbors. Analysis of the parameters using DWT-based features and only the results obtained in the Pareto-front show the use of the *ball tree* and *k-d tree* algorithms to be highly similar, using only two neighbors.

The use of EEG signals from the resting-state with the eyes closed and LOF confirmed that DWT-based features works better, with a TAR of up to $0.997 \pm 0.002$ and TRR of up to $0.950 \pm 0.005,$ with only three EEG channels. The *k-d tree* algorithm, with 2 to 4 neighbors, was the most used for the complete optimization process, as well as the results obtained for the Pareto-front.

The use of OC-SVM can offer good results if the appropriate parameters are chosen. Otherwise, the TAR can decrease substantially. This behavior needs to be further investigated using different feature-extraction methods and compare with the results using different-size datasets. On the other hand, LOF proved to be a robust classifier for creating an EEG-based biometric system, especially using DWT-based features, with the *ball tree* or *k-d tree* algorithms and 2 to 4 neighbors. In the future we will evaluate whether solving the problems related to EMD (Best spline, end effects, mode mixing, etc.) can improve the results presented in this study.

Comparing the results presented in Figs. 3, 7 and 11, one can argue that when using LOF it is more clear to see the most relevant area for choosing a possible set of channels and compare these findings in the future. It is interesting to see that when using resting-state with eyes open or closed did not differ substantially in the channel’s distribution. The localization of most of the relevant channels, i.e the channels that are found in most of the sets, is mainly around channels *F5, T8, T10* and *IZ*, and as it has been shown in Fig. 7, it is more clear when using resting-state with the eyes open. In general, it is possible to mention that most of the channels are localized in the temporal and frontal areas, as well as around the inion, which may be associated with the previous task performed during the data collection. This is an aspect that must be tested in other datasets^39–41^.

One of the purposes of this paper was to prove that resting-state can be used as a paradigm for creating a biometric system in a larger dataset than in our previous work. We have provided a set of experiments where high-density EEG was available during training and testing stages, but for a real-time implementation of the biometric system, we will select only a few of the best channels for designing a new portable headset tailored for this purpose. With the set of experiments and the methods tested for classification and optimization, we have provided a proof-of-concept for a biometric system based on resting-state using a few electrodes, and proved it in a pool with a high number of subjects (109 subjects) compared with our previous work on a smaller dataset. However, with our current results it is not possible to argue that there exists a unique subset of EEG channels or brain regions that works better when creating the biometric system using resting-state. This study is a starting point for analyzing different public and private datasets trying to identify a unique subset of channels that may be used for designing a new portable and easy to use EEG headset that can be tested in real-time, adding new subjects to the system and identifying them with a few electrodes.

It is noticeable the progress in subject identification using EEG signals from different paradigms, but one of the most relevant unsolved problems is that usually the new approaches up to now, are proposed and validated using EEG datasets recorded in well-controlled environments^30,42^. Most of the proposals using high-density EEG signals are recorded with medical-grade sensor systems (Using gel or saline solution for improving the conductivity), which may increase the performance of the methods. However, for practical and portable devices, ease-of-use will be essential and dry electrodes might be offer opportunities here^42^. In general, the analysis and validation in real-life scenarios is necessary. With this, also the best and faster methods will be studied in a more realistic way, and the appropriate and necessary number of trials per subject will be considered^7^.

For certain brain–computer interface (BCI) applications, the problem of recognizing new instances from new sessions has been studied using EEG data from different sessions or adding new instances for calibration. In the case of session-to-session or subject-to-subject transfer, the learning problem has been studied using linear discriminant analysis (LDA) and SVM, based on motor imagery or P300 paradigms^34,43–46^. To adapt the EEG feature space and thus reduce session-to-session variability, we can use a data space adaptation method based on the Kullback-Leibler divergence criterion (Also called relative entropy), aiming to minimize the distribution of differences from the training session to a different session^44^. There is evidence that for certain BCIs, it is possible to use background noise immediately before a new session to improve session-to-session variability using a regularized spatio-temporal filter^45^.

The dataset used in this paper consists of EEG signals from a single session, which limits the experimental configurations and does not allow evaluation of whether we can create models for each subject from a certain session and be able to recognize the subjects or reject them using data from another session. Future steps will center the attention on tackling this problem and analyzing a possible way to use new correctly-classified instances to decrease session-to-session variability, as well as using and comparing current progress in transfer learning, using machine-/deep-learning methods for this problem^16,46^.

Another point to be analyzed in our future work is the comparison with our current proposal and a new way for extracting and selecting the features, looking for improving the TRR and TAR. This can be achieved using a big bag-of-features from the different sub-bands (Possibly from both methods, EMD and DWT) and add additional GA genes for representing these features in the chromosomes and thus select the best features during the optimization process, at the same time of selecting the best channels.

In general, the resting-state has been shown to be a good candidate but there is not yet sufficient research evidence using larger datasets and different stages. Our future efforts will be focused on relevant parameters that can be extracted from the EEG signals of each subject and thus add information for the complete authentication and verification process, such as re-evaluating the accepted subject using multi-class classification, detecting the age-range and sex of the subjects^47^, etc. Additionally, the use of ever larger datasets is still necessary, using EEG data from different sessions and of different lengths, as well as considering fewer instances for training.

## Methods

### Dataset

We used a public dataset that consists of EEG signals of 109 subjects from 64 channels, localized according to the 10–10 international system, with a sample rate of 160 Hz and the recorder using the BCI2000 system. The dataset is part of the physionet project^48^.

Each subject performed two 1-min resting-state runs, one with the eyes open and one with the eyes closed. Then, three 2-min runs were carried out for four different tasks: two motor movement tasks and two imagery tasks^49^. The four types of motor movement and imagery tasks were performed for opening and closing the left or right fist, imagining opening and closing the left or right fist, opening and closing both fists or both feet, and imagining opening and closing both fists or both feet according to the position of a target on the screen (Left, right, top, or bottom).

For this study, we used only the two 1-min baseline runs and created instances of 1 s, obtaining 60 instances of 1 s during the resting-state with the eyes open and 60 instances of 1 s during the resting-state with the eyes closed for each subject.

### Pre-processing and feature extraction

We used the common average reference (CAR) method for improving the signal-to-noise ratio from the EEG signal, which removes the common information from all electrodes that were simultaneously recorded. It can be computed for an EEG channel $V_{i}^{CAR}$, where *i* is the number of the channel, as follows:

1 $$\begin{aligned} V_{i}^{CAR} = V_{i}^{ER}- \frac{1}{n} \sum _{j=1}^{n}V_{j}^{ER} \end{aligned}$$

where $V_{i}^{ER}$ is the potential between the *i*-th electrode and the reference and *n* the number of electrodes.

For comparative purposes, we used two different methods for decomposing the EEG signals and then obtained a set of features for each sub-band.

The first method used was EMD, which is a method that decomposes an EEG signal into a set of finite intrinsic mode functions (IMFs)^50^. Some IMFs that contain limited information may appear in the decomposition, depending on the parameters used in the EMD method (Spline for the interpolation, the method for solving the end effect problem, etc.) and because the numerical procedure is susceptible to errors^51^. These signals show maximum Minkowski distances with respect to the original signal^52^.

Based on the results obtained in our previous research^2,7,12^, we used the closest two IMFs, based on the Minkowski distance, and each IMF was characterized by extracting a set of four features:

1. *Instantaneous energy*, which provides the energy distribution^53^.
2. *Teager energy*, which reflects variations in both the amplitude and frequency of the signal^53,54^.
3. *Higuchi fractal dimension*, which approximates the mean length of the curve using segments of *k* samples and estimates the dimension of a time-varying signal directly in the time domain^55^.
4. *Petrosian fractal dimension* to provide a rapid computation of the fractal dimension of an EEG signal by translating the time series into a binary sequence^56^.

The second method used for decomposing the EEG signals into different frequency bands was DWT. When using DWT, it is necessary to specify the mother function and the levels of decomposition. Here, we use bi-orthogonal 2.2 with three levels of decomposition. We characterized each level of decomposition by extracting the same set of four features described previously.

The process for extracting four features for each selected IMF returns eight features per channel, or 16 features per channel when using DWT. The process is repeated for each channel used to then concatenate them to obtain a single feature vector that represents the EEG signal for each instance.

### Classification

The first classifier that we used for comparative purposes was the well-known OC-SVM, which is an unsupervised algorithm that learns a decision function for outlier detection, classifying new data as similar to or different from that of the training set^57^. The radial basis function (RBF) was used as the kernel and certain important parameters required fitting. As in our previous research^12^, we found that the models based on OC-SVM are very sensitive to *nu* and *gamma* parameters, and they define how well the model learns. The *nu* parameter is an upper bound on the fraction of training errors and a lower bound of the fraction of support vectors that should be in the interval [0, 1]. *Gamma* defines how much influence a single training example has. The larger the *gamma*, the closer other examples must be to be affected and the interval must be greater than 0; normally it is $1/no\_features$.

This work focuses mainly on the use of LOF, which is a density-based unsupervised outlier detection algorithm that defines a degree of being an outlier by calculating the local deviation of a given data point with respect to its surrounding neighborhood. The score assigned to each data point is called the *local outlier factor*^58^. It is based on a concept of local density given by the distance of the k-nearest neighbors. Comparing the local density of a data point with the local densities of its *k* neighbors, it is possible to identify regions with similar density and outliers, which have lower density. The lower the density of a data point, the more likely it is to be identified as an outlier. We can use *brute force*, *ball tree*, or *k-d tree* algorithms for computing the nearest neighbors.

### EEG channel selection

The process for EEG channel selection is critical for the development of a portable low-cost device, and also for analyzing only EEG channels with the relevant information for the discrimination between subjects.

Genetic algorithms (GAs) mimic Darwinian evolution and use biologically inspired operators. Its population is comprised of a set of candidate solutions, each with chromosomes than can be mutated and altered. GAs are normally used to solve complex optimization and search problems^59^. Our approach is based on the non-dominated sorting genetic algorithm (NSGA)^37^, which uses a non-dominated sorting ranking selection method to emphasize good candidates and a niche method to maintain stable sub-populations of good points (Pareto-front). NSGA-II solved certain problems related to the computational complexity, non-elitist approach, and the need to specify a sharing parameter to ensure diversity in a population presented in the first version. NSGA-II also reduced the computational cost from $O(MN^{3})$ to $O(MN^{2})$, where *M* is the number of objectives and *N* the population size. Additionally, the elitist approach was introduced by comparing the current population with the previously found best non-dominated solutions^60^.

We used NSGA-III, as it has been shown to efficiently solve 2- to 15-objective optimization problems^38^ and also because of our previous results^12^. NSGA-III follows the NSGA-II framework but uses a set of predefined reference points that emphasizes population members that are non-dominated, yet close to the supplied set^38,61^. The predefined set of reference points are used to ensure diversity in the obtained solutions.

We used a systematic approach that places points on a normalized hyper-plane that is equally inclined to all objective axes and has an intersection with each axis. For example, in a three-objective optimization problem, the reference points are created on a triangle with apexes at (1, 0, 0), (0, 1, 0), and (0, 0, 1)^61,62^.

Algorithm 1 presents the pseudo-code of the NSGA-III main procedure for a particular generation. The parent population $P_t$ of size *N* is randomly initialized, and then the tournament selection, crossover and mutation operators are applied to create an offspring population $Q_t$. Then, both populations are combined and sorted (According to their domination level) and the best *N* members are selected from the combined population to form the parent population for the next generation. After non-dominated sorting, all acceptable front members and the last front $F_l$ that could not be completely accepted are saved in a set $S_t$. Members in $S_{t}/F_{l}$ are selected for the next generation and the remaining members are selected from $F_l$. NSGA-III use the supplied reference points $Z_r$ for selecting a well distributed set of points. Subsequently, orthogonal distance between a member in $S_t$ and each of the reference lines (Joining the ideal point and a reference point) is computed. The member is then associated with the reference point having the smallest orthogonal distance. Next, the niche count $\rho$ for each reference point, defined as the number of members in $S_{t}/F_{l}$ that are associated with the reference point, is computed for further processing. The reference point having the minimum niche count is identified and the member from the last front $F_l$ that is associated with it is included in the final population. The niche count of the identified reference point is increased by one and the procedure is repeated to fill up population $P_{t+1}$. This niche count as $\rho _j$ for the $j-th$ reference point. A new niche preserving operation is devised by identifying the reference point set $J_{min} = \{j: argmin_j (\rho _j)\}$ having minimum $\rho _j$. In case of multiple such reference points, one ($j^{*}\in J_min$) is chosen at random. If $\rho _{j}^{*} = 0$ (meaning that there is no associated $P_{t+1}$ member to the reference point $j^*$), two scenarios can occur. First, there exists one or more members in front $F_l$ that are already associated with the reference point $j^*$. In this case, the one having the shortest perpendicular distance from the reference line is added to $P_{t+1}$. The count $\rho _{j}^{*}$ is then incremented by one. Second, the front $F_{l}$ does not have any member associated with the reference point $j^*$. In this case, the reference point is excluded from further consideration for the current generation. In the event of $\rho _j^{*} \ge 1$ (Meaning that already one member associated with the reference point exists), a randomly chosen member, if exists, from front $F_l$ that is associated with the reference point $F_l$ is added to $P_{t+1}$. If such a member exists, the count $\rho _{j}^*$ is incremented by one. After $\rho _j$ counts are updated, the procedure is repeated for a total of *K* times to increase the population size of $P_{t+1}$ to *N*^37,38,61,63^. 
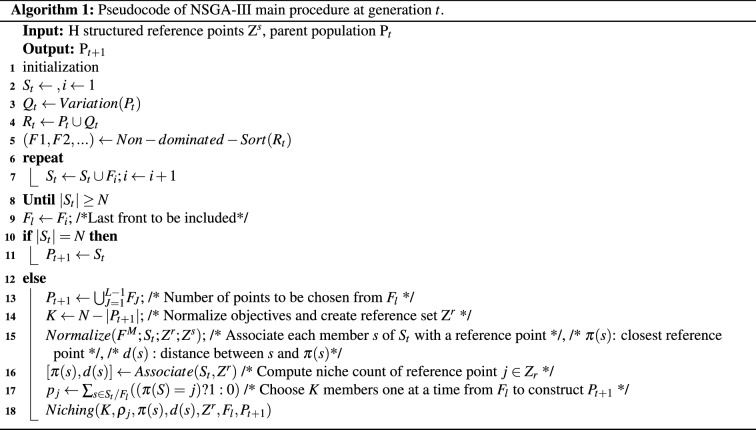


### Definition of the problem to optimize

After the pre-processing and feature extraction stages, we obtain a set of features for each EEG channel. These features can be used to create a model for each subject that can recognize it and reject the rest of the subjects. Our approach is to create a model for each subject with 80% of the instances and use 20% for testing. This requires that certain important parameters be fitted and that the most relevant EEG channels are selected. Thus, the problem is defined as an optimization problem with three unconstrained objectives: minimize the number of necessary EEG channels and maximize the TAR and TRR. The size of each population in each iteration is defined as 20, which was determined experimentally. The termination criterion for the optimization process is defined by the objective space tolerance, which is defined as 0.0001. This criterion is calculated every 10th generation. If optimization is not achieved, the process stops after a maximum of 300 generations.

**Figure 12:**
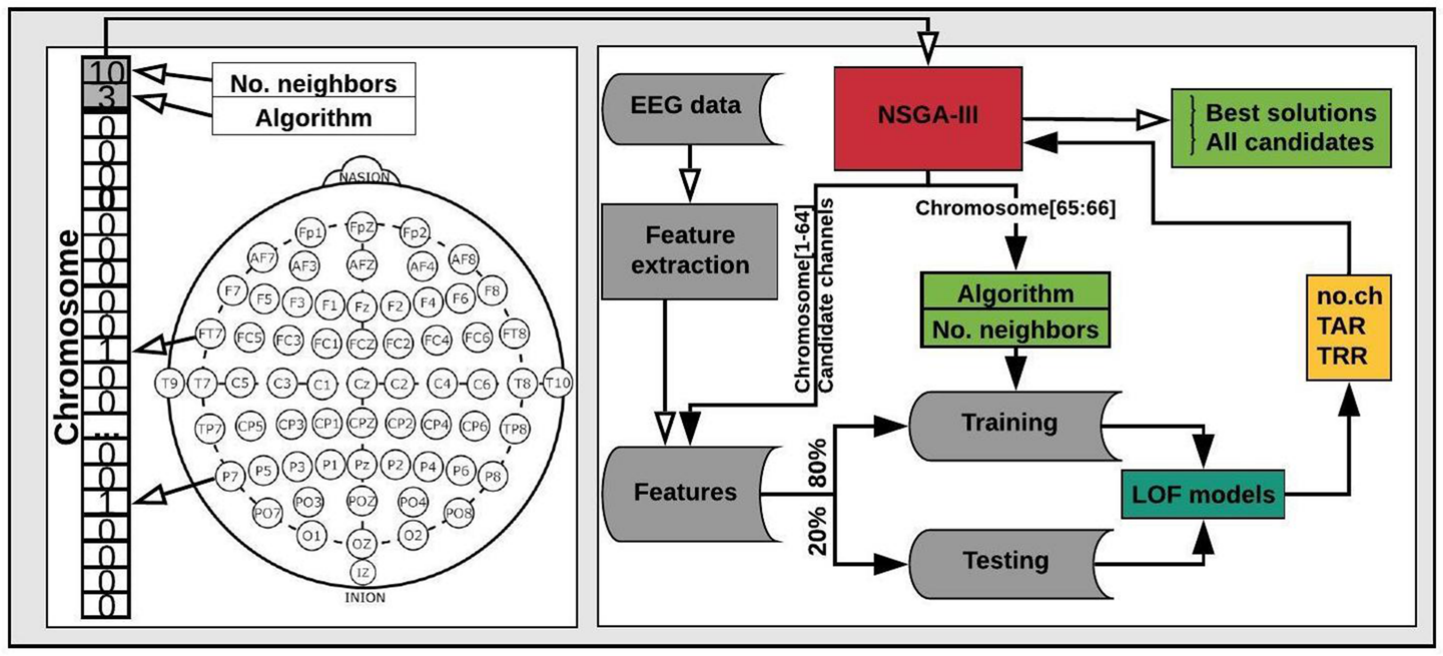
Chromosome representation, and flowchart of the optimization process for EEG channel selection using NSGA-III.

We used 64 binary genes in a chromosome to represent the 64 EEG channels, one gene with integer values to select the algorithm (1: Ball tree, 2: k-d tree, 3: Brute force) and another gene with integer values for selecting the number of neighbors (from 1 to 10, which were proposed experimentally), obtaining thus a chromosome of 66 genes. When using OC-SVM in the optimization process, we used the same 64 genes for representing the EEG channels and two genes with decimal values for selecting the *nu* and *gamma* parameters, similarly to our previous research^12^. The chromosome created to represent the candidate channels in the search space and the flowchart of the complete optimization process using LOF models is illustrated in Fig. 12.

As explained in the feature extraction method, we extracted eight features per channel when using EMD, and 16 when using DWT. The features are organized and stored for iterative use, depending on the channels marked as “1” in the chromosomes. For instance, using EMD-based features, if the chromosome consists of only one gene, the classification process will be performed only with eight features from the channel indicated in the chromosome. The entire process is then performed by NSGA-III, as shown in Fig. 12, which starts creating 20 possible candidates for each generation.

The output for each chromosome for each generation is the number of channels used and the obtained TAR and TRR with the subset of channels in the chromosome. The results are returned to NSGA-III to evaluate each chromosome in the current population, and the new generation of chromosomes is created based on the best candidates found. This process is repeated until the termination criterion or the maximum number of generations is reached.

We performed the experiments for this study on the NTNU IDUN computing cluster^64^. The cluster has more than 70 nodes and 90 GPGPUs. Each node contains two Intel Xeon cores and at least 128 GB of main memory and is connected to an Infiniband network. Half of the nodes are equipped with two or more Nvidia Tesla P100 or V100 GPGPUs. Idun’s storage is provided by two storage arrays and a Lustre parallel distributed file system.

## Data Availability

The dataset used for this study can be found at EEG Motor Movement/Imagery Dataset (EEGMMIDB) https://physionet.org/content/eegmmidb/1.0.0/ from physionet.

## References

[1] Brigham, K. & Kumar, B. V. Subject identification from electroencephalogram (eeg) signals during imagined speech. In *2010 Fourth IEEE International Conference on Biometrics: Theory, Applications and Systems (BTAS)*, 1–8 (IEEE, 2010).

[2] Moctezuma, L. A. & Molinas, M. Eeg-based subjects identification based on biometrics of imagined speech using emd. In *International Conference on Brain Informatics*, 458–467 (Springer, New York, 2018).

[3] Moctezuma LA, Torres-García AA, Villaseñor-Pineda L, Carrillo M (2019). Subjects identification using eeg-recorded imagined speech. Expert Syst. Appl..

[4] Safont, G., Salazar, A., Soriano, A. & Vergara, L. Combination of multiple detectors for eeg based biometric identification/authentication. In *2012 IEEE International Carnahan Conference on Security Technology (ICCST)*, 230–236 (IEEE, 2012).

[5] Fraschini M, Hillebrand A, Demuru M, Didaci L, Marcialis GL (2014). An eeg-based biometric system using eigenvector centrality in resting state brain networks. IEEE Signal Process. Lett..

[6] Kang J-H, Jo YC, Kim S-P (2018). Electroencephalographic feature evaluation for improving personal authentication performance. Neurocomputing.

[7] Moctezuma, L. A. & Molinas, M. Subject identification from low-density eeg-recordings of resting-states: a study of feature extraction and classification. In *Future of Information and Communication Conference*, 830–846 (Springer, New York, 2019).

[8] Di Y (2019). Robustness analysis of identification using resting-state eeg signals. IEEE Access.

[9] Riera A, Soria-Frisch A, Caparrini M, Grau C, Ruffini G (2008). Unobtrusive biometric system based on electroencephalogram analysis. EURASIP J. Adv. Signal Process..

[10] Hu, B., Liu, Q., Zhao, Q., Qi, Y. & Peng, H. A real-time electroencephalogram (eeg) based individual identification interface for mobile security in ubiquitous environment. In *2011 IEEE Asia-Pacific Services Computing Conference*, 436–441 (IEEE, 2011).

[11] Moctezuma, L. A. & Molinas, M. Event-related potential from eeg for a two-step identity authentication system. In *IEEE 17th International Conference on Industrial Informatics (INDIN)* (IEEE, 2019).

[12] Moctezuma, L. A. & Molinas, M. Multi-objective optimization for eeg channel selection and accurate intruder detection in an eeg-based subject identification system. *Sci. Rep.* **10** (2020). 10.1038/s41598-020-62712-6 PMC7125093 32246122

[13] Chen, J., Mao, Z., Yao, W. & Huang, Y. Eeg-based biometric identification with convolutional neural network. *Multimed.Tools Appl.* **1–21** (2019).

[14] Sun Y, Lo FP-W, Lo B (2019). Eeg-based user identification system using 1d-convolutional long short-term memory neural networks. Expert Syst. Appl..

[15] Wilaiprasitporn, T. *et al.* Affective eeg-based person identification using the deep learning approach. *IEEE Transactions on Cognitive and Developmental Systems* (2019).

[16] Özdenizci O, Wang Y, Koike-Akino T, Erdoğmuş D (2019). Adversarial deep learning in eeg biometrics. IEEE Signal Process. Lett..

[17] Davis, P., Creusere, C. D. & Kroger, J. Subject identification based on eeg responses to video stimuli. In *2015 IEEE International Conference on Image Processing (ICIP)*, 1523–1527 (IEEE, 2015).

[18] Schons, T., Moreira, G. J., Silva, P. H., Coelho, V. N. & Luz, E. J. Convolutional network for eeg-based biometric. In *Iberoamerican Congress on Pattern Recognition*, 601–608 (Springer, New York, 2017).

[19] Zhang X (2018). Mindid: Person identification from brain waves through attention-based recurrent neural network. Proc. ACM.

[20] Jin, L., Chang, J. & Kim, E. Eeg-based user identification using channel-wise features. In *Asian Conference on Pattern Recognition*, 750–762 (Springer, New York, 2019).

[21] La Rocca D (2014). Human brain distinctiveness based on eeg spectral coherence connectivity. IEEE Trans. Biomed. Eng..

[22] Crobe A, Demuru M, Didaci L, Marcialis GL, Fraschini M (2016). Minimum spanning tree and k-core decomposition as measure of subject-specific eeg traits. Biomed. Phys. Eng. Express.

[23] Garau, M., Fraschini, M., Didaci, L. & Marcialis, G. L. Experimental results on multi-modal fusion of eeg-based personal verification algorithms. In *2016 International Conference on Biometrics (ICB)*, 1–6 (IEEE, 2016).

[24] Thomas, K. P. & Vinod, A. P. Biometric identification of persons using sample entropy features of eeg during rest state. In *2016 IEEE International Conference on Systems, Man, and Cybernetics (SMC)*, 003487–003492 (IEEE, 2016).

[25] Thomas, K. P. & Vinod, A. P. Utilizing individual alpha frequency and delta band power in eeg based biometric recognition. In *2016 IEEE International Conference on Systems, Man, and Cybernetics (SMC)*, 004787–004791 (IEEE, 2016).

[26] Barra S, Casanova A, Fraschini M, Nappi M (2017). Fusion of physiological measures for multimodal biometric systems. Multimed. Tools Appl..

[27] Yang S, Deravi F, Hoque S (2018). Task sensitivity in eeg biometric recognition. Pattern Anal. Appl..

[28] Campisi P, La Rocca D (2014). Brain waves for automatic biometric-based user recognition. IEEE Trans. Inf. Forensics Secur..

[29] Abo-Zahhad M, Ahmed SM, Abbas SN (2015). State-of-the-art methods and future perspectives for personal recognition based on electroencephalogram signals. IET Biometr..

[30] Bidgoly, A. J., Bidgoly, H. J. & Arezoumand, Z. A survey on methods and challenges in eeg based authentication. *Comput. Secur.* **101788** (2020).

[31] Gui Q, Ruiz-Blondet MV, Laszlo S, Jin Z (2019). A survey on brain biometrics. ACM Comput. Surv..

[32] Altahat, S., Wagner, M. & Marroquin, E. M. Robust electroencephalogram channel set for person authentication. In *2015 IEEE International Conference on Acoustics, Speech and Signal Processing (ICASSP)*, 997–1001 (IEEE, 2015).

[33] Rodrigues D, Silva GF, Papa JP, Marana AN, Yang X-S (2016). Eeg-based person identification through binary flower pollination algorithm. Expert Syst. Appl..

[34] Lotte F (2018). A review of classification algorithms for eeg-based brain-computer interfaces: a 10 year update. J. Neural Eng..

[35] Koelstra S (2011). Deap: a database for emotion analysis; using physiological signals. IEEE Trans. Affect. Comput..

[36] Mao, Z., Yao, W. X. & Huang, Y. Eeg-based biometric identification with deep learning. In *2017 8th International IEEE/EMBS Conference on Neural Engineering (NER)*, 609–612 (IEEE, 2017).

[37] Srinivas N, Deb K (1994). Muiltiobjective optimization using nondominated sorting in genetic algorithms. Evol. Comput..

[38] Deb K, Jain H (2013). An evolutionary many-objective optimization algorithm using reference-point-based nondominated sorting approach, part i: solving problems with box constraints. IEEE Trans. Evol. Comput..

[39] Mizuguchi N (2013). Brain activity during motor imagery of an action with an object: a functional magnetic resonance imaging study. Neurosci. Res..

[40] Miller KJ (2010). Cortical activity during motor execution, motor imagery, and imagery-based online feedback. Proc. Natl. Acad. Sci..

[41] Taube W (2015). Brain activity during observation and motor imagery of different balance tasks: an fmri study. Cortex.

[42] Yang S, Deravi F (2017). On the usability of electroencephalographic signals for biometric recognition: a survey. IEEE Trans. Hum. Mach. Syst..

[43] Pan SJ, Yang Q (2009). A survey on transfer learning. IEEE Trans. Knowl. Data Eng..

[44] Arvaneh M, Guan C, Ang KK, Quek C (2013). Eeg data space adaptation to reduce intersession nonstationarity in brain-computer interface. Neural Comput..

[45] Cho H, Ahn M, Kim K, Jun SC (2015). Increasing session-to-session transfer in a brain-computer interface with on-site background noise acquisition. J. Neural Eng..

[46] Li F (2020). Transfer learning algorithm of p300-eeg signal based on xdawn spatial filter and riemannian geometry classifier. Appl. Sci..

[47] Moctezuma, L. A. & Molinas, M. Sex differences observed in a study of eeg of linguistic activity and resting-state: Exploring optimal eeg channel configurations. In *2019 7th International Winter Conference on Brain-Computer Interface (BCI)*, 1–6 (IEEE, 2019).

[48] Goldberger AL (2000). Physiobank, physiotoolkit, and physionet: components of a new research resource for complex physiologic signals. Circulation.

[49] Schalk G, McFarland DJ, Hinterberger T, Birbaumer N, Wolpaw JR (2004). Bci 2000: a general-purpose brain-computer interface (bci) system. IEEE Trans. Biomed. Eng..

[50] Huang NE (1998). The empirical mode decomposition and the hilbert spectrum for nonlinear and non-stationary time series analysis. Proc. R. Soc. Lond. A.

[51] Rilling, G., Flandrin, P., Goncalves, P. *et al.* On empirical mode decomposition and its algorithms. In *IEEE-EURASIP Workshop on Nonlinear Signal and Image Processing*, vol. 3, 8–11 (NSIP-03, Grado (I), 2003).

[52] Boutana, D., Benidir, M. & Barkat, B. On the selection of intrinsic mode function in emd method: application on heart sound signal. In *2010 3rd International Symposium on Applied Sciences in Biomedical and Communication Technologies (ISABEL 2010)*, 1–5 (IEEE, 2010).

[53] Didiot E, Illina I, Fohr D, Mella O (2010). A wavelet-based parameterization for speech/music discrimination. Comput. Speech Lang..

[54] Jabloun, F. & Cetin, A. E. The teager energy based feature parameters for robust speech recognition in car noise. In *1999 IEEE International Conference on Acoustics, Speech, and Signal Processing. Proceedings. ICASSP99 (Cat. No. 99CH36258)*, vol. 1, 273–276 (IEEE, 1999).

[55] Higuchi T (1988). Approach to an irregular time series on the basis of the fractal theory. Physica D.

[56] Petrosian, A. Kolmogorov complexity of finite sequences and recognition of different preictal eeg patterns. In *Proceedings Eighth IEEE Symposium on Computer-Based Medical Systems*, 212–217 (IEEE, 1995).

[57] Schölkopf B, Smola AJ, Williamson RC, Bartlett PL (2000). New support vector algorithms. Neural Comput..

[58] Breunig, M. M., Kriegel, H.-P., Ng, R. T. & Sander, J. Lof: identifying density-based local outliers. In *Proceedings of the 2000 ACM SIGMOD International Conference on Management of Data*, 93–104 (2000).

[59] Chugh T, Sindhya K, Hakanen J, Miettinen K (2019). A survey on handling computationally expensive multiobjective optimization problems with evolutionary algorithms. Soft Comput..

[60] Deb K, Pratap A, Agarwal S, Meyarivan T (2002). A fast and elitist multiobjective genetic algorithm: Nsga-ii. IEEE Trans. Evol. Comput..

[61] Jain H, Deb K (2013). An evolutionary many-objective optimization algorithm using reference-point based nondominated sorting approach, part ii: handling constraints and extending to an adaptive approach. IEEE Trans. Evol. Comput..

[62] Das I, Dennis JE (1998). Normal-boundary intersection: a new method for generating the pareto surface in nonlinear multicriteria optimization problems. SIAM J. Optim..

[63] Mkaouer W (2015). Many-objective software remodularization using nsga-iii. ACM Trans. Softw. Eng. Methodol. (TOSEM).

[64] Själander, M., Jahre, M., Tufte, G. & Reissmann, N. EPIC: An energy-efficient, high-performance GPGPU computing research infrastructure (2019). arXiv:1912.05848.

